# Clinical Evaluation of an Immunochromatographic-Based IgM/IgG Antibody Assay (GenBody™ COVI040) for Detection of Antibody Seroconversion in Patients with SARS-CoV-2 Infection

**DOI:** 10.3390/diagnostics11030537

**Published:** 2021-03-17

**Authors:** Doyeong Kim, Jihoo Lee, Jyotiranjan Bal, Chom-Kyu Chong, Jong Ho Lee, Hyun Park

**Affiliations:** 1 GenBody Inc., Cheonan 31116, Korea; dykim@genbody.co.kr (D.K.); jhlee@genbody.co.kr (J.L.); 2 Zoonosis Research Center, Department of Infection Biology, School of Medicine, Wonkwang University, Iksan 570-749, Korea; jyoti_micro@yahoo.co.in; 3 Department of Laboratory Medicine, Yeungnam University College of Medicine, Daegu 42415, Korea

**Keywords:** SARS-CoV-2, sensitivity, specificity, COVID-19, diagnostic accuracy study, immunochromatography, seroconversion, immunoglobulin G, immunoglobulin M

## Abstract

There is a need for accurate diagnostic tests for severe acute respiratory syndrome coronavirus 2 (SARS-CoV-2), the cause of coronavirus disease (COVID-19). This study aimed to evaluate the diagnostic accuracy of an immunochromatography-based immunoglobulin G (IgG)/immunoglobulin M (IgM) antibody assay (GenBody™ COVI040) for detecting SARS-CoV-2 antibody seroconversion in COVID-19 patients. A total of 130 samples, serially collected from patients with confirmed COVID-19, and 100 negative control samples were tested for anti-SARS-CoV-2 IgM and IgG using the GenBody™ COVI040 assay following the South Korean Ministry of Food and Drug Safety guidelines on the review and approval of in vitro diagnostic devices for COVID-19. Reverse-transcription polymerase chain reaction results were used as the comparator. The overall sensitivity of the GenBody™ COVI040 assay was 97.69% (95% confidence interval (CI): 93.40–99.52%). The sensitivity of the assay increased with time post symptom onset (PSO) (sensitivity ≤6 days PSO: 78.57%, 95% CI: 49.20–95.34%; sensitivity 7–13 days PSO: 100%, 95% CI: 87.23–100%; and sensitivity ≥14 days PSO: 100%, 95% CI: 95.94–100%). The specificity of the assay was 100% (95% CI: 96.38–100%). The GenBody™ COVI040 assay showed high sensitivity and specificity, making it a promising diagnostic test to monitor COVID-19.

## 1. Introduction

The coronavirus disease (COVID-19) pandemic, which started in late 2019 in Wuhan, China [1], continues to be a major threat for the global population (World Health Organization (WHO)). As of 27 December 2020, a cumulative total of over 79.2 million cases and over 1.7 million deaths were reported since the start of the pandemic, raising serious concerns about global healthcare and the global economy (WHO). With vaccines yet to be rolled out and a lack of effective treatments, most countries have relied on diagnosis and contact tracing followed by strict quarantine in an attempt to control the spread of the virus.

The cause of COVID-19, severe acute respiratory syndrome coronavirus 2 (SARS-CoV-2), is a single stranded positive-sense RNA virus of 29.9 kb [2,3], consisting of genes encoding, among others, spike (S), envelope (E), membrane (M), and nucleocapsid (N) structural proteins, of which S and N, being highly immunogenic, are widely used as targets in molecular and serological diagnostic assays [4].

Most premier medical bodies throughout the world validate nucleic acid amplification tests using real time reverse-transcription polymerase chain reaction (RT-PCR) of nasopharyngeal and oropharyngeal swabs as the primary method of diagnosis of SARS-CoV-2 [5]. However, RT-PCR has many limitations including high cost; required expertise; and, most importantly, the dependence of the result on the timing of sample collection, with viral load declining after the first week post symptom onset (PSO) [6,7]. Detection of antibody seroconversion that can detect virus-specific antibodies despite negative nucleic acid tests is a promising alternative to RT-PCR for diagnosis, monitoring the immune response, determining infection rates, and identification of potential serum donors of SARS-CoV-2 antibodies for immunotherapy [8,9]. Seroconversion, the moment when antibody levels become detectable in the blood, takes place a few days after the viral load has peaked [6]. Thus, a knowledge of the antibody response kinetics is necessary for the proper application of serological assays. Immunoglobulin G (IgG) and immunoglobulin M (IgM) are promising biomarkers for monitoring the humoral immune responses to SARS-CoV-2 infection [10,11,12].

Among the various types of serological tests, rapid diagnostic tests (RDTs) based on antibody detection on nitrocellulose paper are popular owing to their ease of performance, portability, rapid turnaround time, and point-of-care (POC) applications. Regarding SARS-CoV-2 detection, RDTs involving lateral flow immunoassays (LFIA) have been reported to have exhibited sensitivity and specificity of 97.5% and 95.2%, respectively, which was at par with that of various other serological tests including enzyme linked immunosorbent assay (ELISA) and chemiluminescence immunoassay (CLIA) [13]. RDTs showed highest concordance with specificities of 98% and sensitivities of 87–100% in comparison with immunofluorescence assay and automated ELISA [14]. Although several serological assays had been developed for the detection of SARS-CoV-2 antibodies, the published evidence of performance is limited, with few of them having received emergency approval, while many are still awaiting clinical evaluation.

This study aimed to assess the diagnostic sensitivity and specificity of an immunochromatography-based IgM/IgG antibody assay (GenBody™ COVI040) for detecting SARS-CoV-2 antibody seroconversion in patients with COVID 19 according to the time post symptom onset (PSO).

## 2. Materials and Methods

### 2.1. Ethics Statement

This study was conducted according to International Standards of Good Clinical Practice at Yeungnam University Medical Center (YUMC), South Korea and submitted to a properly constituted institutional review board (IRB), in agreement with local legal and ethical standards for formal approval of candidate diagnostic tests (IRB No.: YUMC 2020-06-057/2020, approval date: 25 June 2020).

### 2.2. Reagents Used in the Kit

#### 2.2.1. Preparation of SARS-CoV-2 Nucleocapsid Protein (NP) Antigen

SARS-CoV-2 NP DNA was synthesized by Bioneer, South Korea. The NP gene was amplified by PCR with a forward primer (5′-TTT GGA TCC AGC GAT AAT GGA CCG CAG AAT) and a reverse primer (5′-TTT GTC GAC GGC CTG AGT TGA ATC AGC ACT GCT). A 1254 bp section of the NP gene was successfully amplified and subcloned into pET expression vectors (Novagen, Merck Millipore, Darmstadt, Germany), which can express the recombinant protein in *Escherichia coli*. Gene cloning was confirmed through sequencing analysis. When the OD600 reached 0.6–0.8 at 37 °C, induction was performed using 0.1 mM isopropyl β-d-1-thiogalactopyranoside (IPTG). After incubating the culture medium at 24 °C for 18 h after induction, the culture medium was harvested and the protein was purified.

#### 2.2.2. Preparation of SARS-CoV-2 S Antigen

Codon optimized SARS-CoV-2 S glycoprotein was synthesized and cloned into Drosophila cell expression vector pMT/BiP/V5-His vector between KpnI and XhoI sites. Drosophila Schneider’s line 2 (S2) cells were maintained in Schneider’s Drosophila medium at 28 °C. For transfection, S2 cells were sub-cultured 3–5 days before transfection, and 1 × 10^6^ cells in 3 mL medium were aliquoted into a 35 mm plate. For selection of stable cell lines, cells were transfected with 19 ug of DNA and 1 µg of pCoBlast using CaCl_2_. After 24 h, CaCl_2_ was removed and replaced with complete medium. After 2 days at 28 °C, cells were resuspended in growth medium containing blasticidin. Selective mediums were replaced every 5 days until resistant colonies appeared. When the cells reached a density of 6 × 10^6^ cells/mL, cells were transferred for large-scale expression. For induction of the target protein, 500 µM of copper sulfate was added. After 1 week of induction, culture medium was harvested and protein purified.

### 2.3. Preparation of RDT Strips

**Figure 1:**
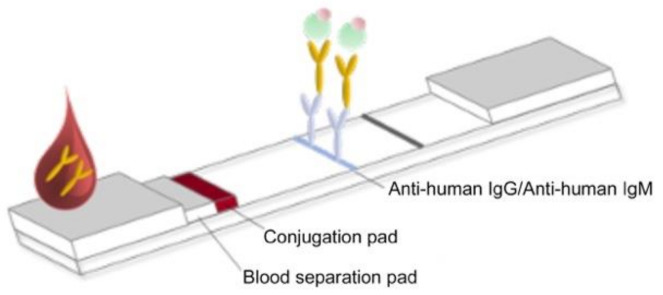
Schematic representation of the test strip for measuring anti-severe acute respiratory syndrome coronavirus 2 (SARS-CoV-2) immunoglobulin G (IgG) and immunoglobulin M (IgM).

Colloidal gold particles were prepared as previously described (Frens, 1973). HAuCl4 (0.02%) was boiled in a beaker and 0.2% sodium citrate was added while stirring constantly. When the solution turned a wine-red color, it was boiled for another 5 min and stirred for 10 min without boiling. The colloidal gold solution was stored in the dark at 4 °C until use. The recombinant SARS-CoV-2 nucleocapsid protein (NP) (1 mg) and recombinant SARS-CoV-2 (spike) S1 protein were respectively conjugated with prepared colloidal gold particles (100 mL). The antigen–gold conjugates were precipitated by centrifugation and dissolved with phosphate-buffered saline containing 0.1% bovine serum albumin (BSA) to adjust the OD450 to 10. The conjugates were then treated on a glass fiber and dried to prepare the conjugator pad. The mouse mAbs against human IgG and human IgM were dispensed and immobilized at the appropriate positions on a nitrocellulose membrane (2.5 mg/mL). Goat anti-mouse IgG (1 mg/mL) (Arista Biologicals Inc., Allentown, PA, USA) was dispensed and immobilized on the control line of the membrane. The buffer pad was prepared by treating cellulose paper (Grade 319; Ahlstrom Inc., Alpharetta, GA, USA) with 0.1 M carbonate buffer (pH 9.0). The absorbance pad consisted of untreated cotton paper. All pads were partially overlapped to enable the migration of the sample and buffer solution along the strip (Figure 1).

### 2.4. Specimen Collection

Serum samples collected from patients who visited the hospital with COVID-19 symptoms and had SARS-CoV-2 infection confirmed using Real-Q 2019-nCoV Detection Kit (BioSewoom Inc., Seoul, Korea) and AllplexTM 2019-nCoV Assay (Seegene Inc., Seoul, Korea) were used as positive samples. A total of 15 µL of each serum sample was collected within 6 months of storage at −20 °C for further analysis. Positive samples included information such as the symptom onset date, confirmation date, and sample collection date. Samples collected ≥14 days PSO were excluded. Samples with severe hemolysis, incomplete information, suspected contamination, a volume <0.5 µL, or stored at temperatures above −70 °C were excluded.

### 2.5. Calculation of the Sample Size

Based on the guidelines of the review and approval of in vitro diagnostic devices for COVID-19 prescribed by the Republic of Korea Ministry of Food and Drug Safety, the equation for the sample size for determining clinical sensitivity and specificity was calculated using the following formula:

$$n\;=\;\frac{\left(\frac{𝑍\alpha }{2}+𝑍\beta \right)2\;\times \;P1\left(1-P1\right)}{\left(P1-P0\right)2}$$

*P*1: Estimated sensitivity or specificity of the diagnostic;

*P*0: Lower limit of the 95% confidence interval (CI) of the target sensitivity or specificity;

*𝑍α*/2: t value of type 1 error (*α*);

*𝑍β*: t value of type 2 error (*β*).

### 2.6. Methods for Using the Diagnostic Test

**Figure 2:**
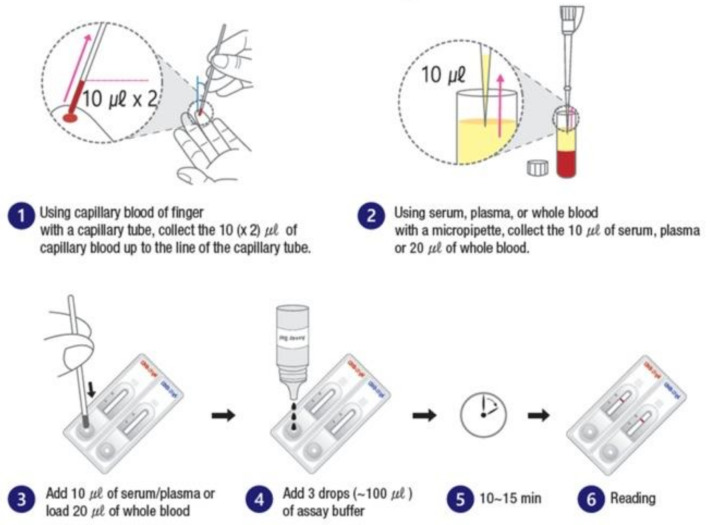
Schematic representation of the assay procedure.

All specimens, test strips, and test reagents were acclimatized to room temperature for 15–30 min before the test. When using a capillary tube, serum was collected up to the black line (10 µL) of the capillary tube; otherwise, 10 µL of plasma and 20 µL of whole blood were added dropwise to the drop site. This was followed by the addition of three drops (approximately 100 µL) of the sample developing solution to the drop site. The development of bands was observed after 10–15 min (Figure 2). Results read ≥20 min were considered invalid.

### 2.7. Interpretation of the Results

**Figure 3:**
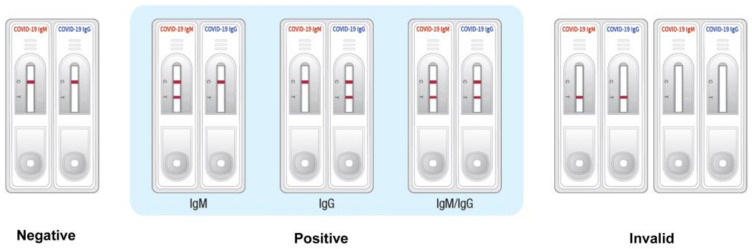
Interpretation of the test results of the rapid diagnostic test (RDT) strip.

The interpretation of the test results is depicted in Figure 3. If colored bands appeared only on the control line (C), the test was interpreted as negative for SARS-CoV-2-specific IgM and IgG. If COVID-19 was suspected, the sample was retested after 3–5 days. If the colored band appeared on both the test line (T) and the control line (C) of the left test kit, then it was considered to be IgM positive, and if the colored band appeared on both the test line (T) and the control line (C) of the right test kit, it was considered to be IgG positive. Both IgM and IgG were considered positive if colored bands appeared on the test lines (T) and control lines (C) of both test kits. If a colored band did not appear on the control line (C) after 10 min, the test result was considered invalid. If the test result was invalid, the patient’s sample was inspected using a new device.

### 2.8. Evaluation Criteria

According to the guidelines of the Ministry of Food and Drug Safety, the criteria for the evaluation of the clinical sensitivity and specificity of the test device using the obtained human blood are as follows: (1) clinical sensitivity: ≥80% (lower limit of 95% CI ≥ 70%); and (2) clinical specificity: ≥95% (lower limit of 95% CI ≥ 90%). The seroconversion pattern was determined using samples collected from the same patient consecutively.

Clinical sensitivity (%) = true positive/(true positive + false negative) × 100

If at least one of IgM and IgG is positive, it is considered positive and reflected in clinical sensitivity.

Clinical specificity (%) = true negative/(false positive + true negative) × 100

When both IgM and IgG are negative, they were considered negative and the result was used in the calculation of clinical specificity. The lower limits of the 95% CIs of clinical sensitivity and specificity were required to be higher than the lower limit of the 95% CI for clinical efficacy.

## 3. Results

### 3.1. Expression and Purification of Recombinant SARS-CoV-2 NP and SARS-CoV-2 S

**Figure 4:**
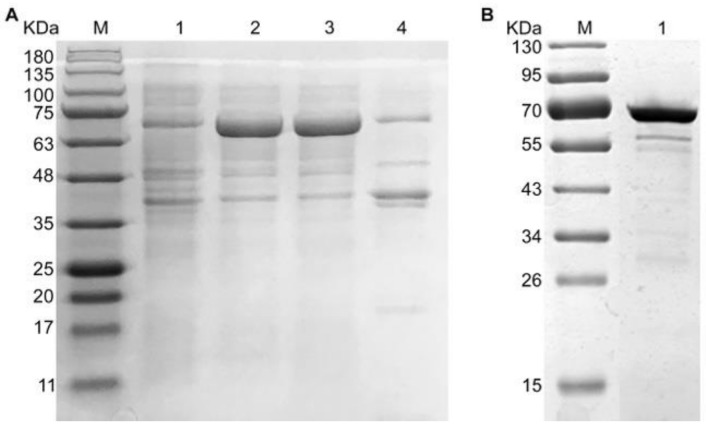
Expression and purification of recombinant NP of SARS-CoV-2 in *E. coli*. (**A**) Detection of recombinant NP through SDS-PAGE under different conditions. M: marker, 1: before IPTG induction, 2: after isopropyl β-d-1-thiogalactopyranoside (IPTG) induction, 3: supernatant, 4: insoluble fraction. (**B**) Purified recombinant NP. M: marker, 1: purified recombinant NP.

The target protein recombinant SARS-CoV-2 NP was soluble expressed in *E. coli* (Figure 4A) and successfully purified to homogeneity using metal affinity chromatography (Ni-NTA) (Figure 4B). It was detected approximately at 70KDa (Figure 4).

**Figure 5:**
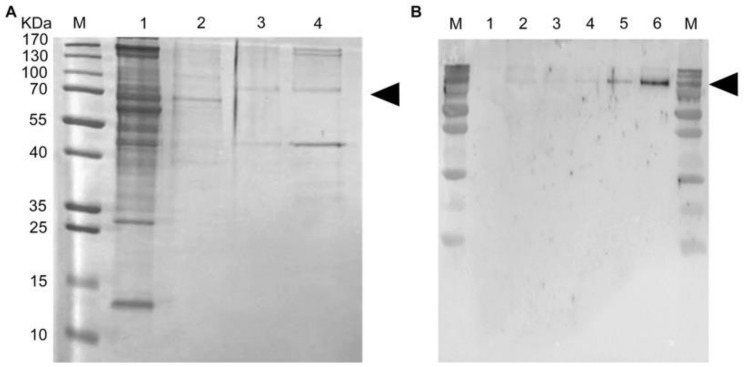
Purification and confirmation of recombinant S protein of SARS-CoV-2 expressed in Drosophila Schneider’s line 2 (S2) cells. (**A**) Purification of recombinant S under different conditions. M: marker, 1: 30 mM imidazole, 2: 60 mM imidazole, 3: 80 mM imidazole, 4: 250 mM imidazole. (**B**) Western blot analysis of purified S protein. M: marker; 1: negative control; 2: transient expression of recombinant S in Drosophila expression system; 3–6: expression of recombinant S1 in stable cell lines, 1 µL, 2 µL, 5 µL, and 10 µL.

Furthermore, the second target protein recombinant SARS-CoV-2 S was successfully expressed in Drosophila Schneider’s line 2 (S2) cells (Figure 5A) and successfully purified to homogeneity using metal affinity chromatography (Ni-NTA) (Figure 5B). 

### 3.2. Clinical Specimen Validation

Overall, 230 clinical specimens were collected (Table 1) for the clinical evaluation of the GenBody™ COVI040 assay. Of the 230 specimens, 100 were negative and 130 were positive for SARS-CoV-2 based on the RT-PCR results. Of the 130 positive samples, 14 were collected <7 days after PSO, 27 were collected 7–13 days after PSO, and the remaining 89 samples were collected ≥14 days after PSO.

### 3.3. Clinical Sensitivity and Specificity of the GenBody™ COVI040 Assay

The primary diagnostic accuracy results estimated the clinical sensitivity of the GenBody™ COVI040 assay to be 97.69% (95% CI: 93.40–99.52%), while the clinical specificity was estimated to be 100% (95% CI: 96.38–100%), as shown in Table 2.

#### 3.3.1. Clinical Sensitivity and Specificity of IgM/IgG Detection

Clinical sensitivity for the detection of IgM only using GenBody™ COVI040 assay was estimated to be 82.31% (95% CI: 74.65–88.44%), while the clinical specificity was estimated to be 100% (95% CI: 96.38–100%), as shown in Table 3.

The clinical sensitivity for the detection of IgG only using the IgM/IgG antibody assay (GenBody™ COVI040) was estimated to be 97.69% (95% CI: 93.40–99.52%), while the clinical specificity was estimated to 100% (95% CI: 96.38–100%), as shown in Table 4.

#### 3.3.2. Clinical Sensitivity and Specificity Based on Days PSO

The sensitivity and specificity of the GenBody COVID-19 kit estimated from testing samples collected over a period of >14 days PSO are summarized in Table 5. The overall sensitivity was 78.57% (95% CI: 49.2–95.34%) using samples collected <7 days PSO, and increased with increasing days PSO. The sensitivity increased to 100% (95% CI: 87.23–100%) and 100.0% (95% CI: 95.94–100%) in samples collected ≥7 days and ≥14 days PSO, respectively.

### 3.4. Limit of Detection by the GenBody COVID-19 Kit

The limit of detection by the GenBody COVID-19 kit was estimated through serially diluted serum samples, as shown in Supplementary Table S1. IgM was detected up to a 10−2 dilution, whereas IgG was detected up to 10−4 dilution, confirming the high sensitivity of the GenBody COVID-19 diagnostic kit.

### 3.5. Evaluation of Seroconversion

Seroconversion during the 24 days PSO was evaluated using samples collected consecutively from five patients (Table 6). IgM could not be detected on days 0, 15, and 20 days PSO, whereas IgG could not be detected on 0 and 1 days PSO. All other specimens showed positive results.

## 4. Discussion

The rising number of cases of COVID-19 necessitates large-scale population-based serological testing to determine the seroprevalence of antibodies against SARS-COV-2 and, thereby, to evaluate the potential herd immunity and the association between antibody positivity and immunity [12,15]. Multiple diagnostic tests for SARS-CoV-2 antibody testing have been developed, but it is necessary for each test to undergo a thorough clinical evaluation according to standards and regulations to ensure adequate test performance for their intended clinical use. In this study, we evaluated the performance of an immunochromatographic based IgM/IgG antibody assay, GenBody™ COVI040, following the norms and guidelines on the review and approval of in vitro diagnostic devices for COVID 19, as recommended by the South Korean Ministry of Food and Drug Safety. This rapid test has the sensitivity to detect both nucleocapsid-associated and spike protein-associated antibodies and, thereby, has a higher diagnostic accuracy than some other serological assays. The diagnostic accuracy evaluation estimated the clinical sensitivity to be 97.69%, and clinical specificity to be 100%, with a sample size of 230 positive and 100 negative samples, with the result previously confirmed using SARS-CoV-2 RT-PCR assays. Previous studies evaluating the clinical efficiency of antibody tests used a limited number of confirmed infected samples as well as negative samples [16,17]. These tests were reported to have a low clinical sensitivity from 45% to 65%, with a clinical specificity between 87.3% and 100% [16], in contrast to the higher sensitivity and specificity of the current study.

Serum analysis of individuals affected by COVID 19 confirmed the interaction of host neutralizing antibodies with S and N proteins, thus increased efficiency is predicted in serological tests involving S and N proteins [18]. ELISA kit based on the recombinant full-length SARS-CoV-2 S1 protein exhibited specificity of 97.5%, as examined against a total of 412 normal human samples, and sensitivity of 97.1% against 69 samples from hospitalized and/or recovered COVID-19 patients [19].

The clinical sensitivity of detection of previously RT-PCR diagnosed positive samples increased with increasing days PSO, revealing access to seroprevalence monitoring. These findings are comparable to those of previous studies evaluating the performance of other immunochromatography-based assays [17,20,21].

RDTs are intended to complement molecular testing (more accurate after day 5), sero-epidemiological investigations, diagnostic testing of asymptomatic or weakly symptomatic individuals, or individuals within the latent period [22,23]. The investigated GenBody™ COVI040 assay provided readily interpretable results, comparable to RT-PCR results, within 10–15 min, confirming earlier reports. As it does not require any sophisticated instrumentation or expensive materials, it is recommended for use in large-scale sero-epidemiological field investigations outside the laboratory. It further enables POC sample collection as an alternative to painful oropharyngeal and nasopharyngeal specimen collection for RT-PCR testing.

Although many treatment strategies have been developed to manage and treat individuals with COVID-19, early prevention of human-to-human transmission of SARS-CoV-2 by asymptomatic patients remains a challenge. This highlights the importance of serological tests as an alternative to molecular tests. IgM and IgG antibodies are sensitive biomarkers for monitoring the seroprevalence of SARS-CoV-2 [10,24]. Even with a steady decline in SARS-CoV-2 viral load, IgG/IgM seroconversion can be detected in asymptomatic individuals and individuals with mild disease [9]. IgM and IgG seroconversion in individuals with SARS-CoV-2 infection can occur simultaneously or sequentially [6,25]. IgM antibody levels usually peak as early as the third week PSO, and for this reason, immunochromatography-based lateral flow immunoassays that can simultaneously measure IgM and IgG are considered more useful for detecting seroconversion [26]. This study confirms the utility of this diagnostic kit for serological monitoring as it detects both IgG and IgM. The results of this evaluation confirmed that IgM could not be detected on days 0, 15, and 20 PSO, whereas IgG could not be detected on days 0 and 1 PSO, while the combination of both IgM and IgG had higher sensitivity with increasing time PSO. Thus, these findings confirm that GenBody™ COVI040 is an efficient diagnostic tool for serological monitoring of SARS-CoV-2 infection.

## 5. Conclusions

GenBody™ (COVI040) assay was evaluated for its clinical sensitivity and specificity for detection of SARS-CoV-2 IgM and IgG antibodies in human serum following prescribed standards and regulations. It is intended for bulk screening, sero-epidemiological investigations, and testing of asymptomatic or weakly symptomatic individuals and those in the latent period, and plays a complementary role to molecular testing. Thus, this study supports the use of GenBody™ (COVI040) assay as a reliable diagnostic test for SARS-CoV-2 IgM and IgG antibody detection.

## Figures and Tables

**Table 1:** Sample size and timing of specimen collection for the clinical evaluation.

	Positive				Negative	Total
Days post symptom onset (PSO)	≤7	7–13	≥14	Total		
Number of Samples	14	27	89	130	100	230

**Table 2:** Results of the tests using the GenBody™ COVI040 assay.

Evaluation Results of Test Equipment (GenBody COVID-19 Kit) IgM/IgG	Confirmed Results through RT-PCR		Total
	Positive	Negative	
Positive	127	0	127
Negative	3	100	103
Total	130	100	230

Clinical sensitivity: (127/130) × 100 = 97.69% (95% confidence interval (CI): 93.40–99.52%). Clinical specificity: (100/100) × 100 = 100% (95% CI: 96.38–100%). RT-PCR, SARS-CoV-2 reverse-transcription polymerase chain reaction.

**Table 3:** IgM antibody test results using the GenBody™ COVI040 assay.

Evaluation Results of Test Equipment (GenBody COVID-19 kit) IgM	Confirmed Results through RT-PCR		Total
	Positive	Negative	
Positive	107	0	107
Negative	23	100	123
Total	130	100	230

Clinical sensitivity: (107/130) × 100 = 82.31% (95% CI: 74.65–88.44%). Clinical specificity: (100/100) × 100 = 100% (95% CI: 96.38–100%). RT-PCR, SARS-CoV-2 reverse-transcription polymerase chain reaction.

**Table 4:** IgG antibody test results using the GenBody™ COVI040 assay.

Evaluation Results of Test Equipment (GenBody COVID-19 Kit) IgG	Confirmed RESULTS through RT-PCR		Total
	Positive	Negative	
Positive	127	0	127
Negative	3	100	103
Total	130	100	230

Clinical sensitivity: (127/130) × 100 = 97.69% (95% CI: 93.40–99.52%). Clinical specificity: (100/100) × 100 = 100.00% (95% CI: 96.38–100.00%). RT-PCR, SARS-CoV-2 reverse-transcription polymerase chain reaction.

**Table 5:** Clinical sensitivity and specificity based on days PSO.

Evaluation Results of Test Equipment (GenBody COVID-19 Kit)	Confirmed Results through RT-PCR Positive								
	0–6 Days PSO			7–13 Days PSO			≥14 Days PSO		
	IgM/IgG	IgM	IgG	IgM/IgG	IgM	IgG	IgM/IgG	IgM	IgG
Positive	11	10	11	27	23	27	89	74	89
Negative	03	04	03	00	04	00	00	15	00
Total	14	14	14	27	27	27	89	89	89
Sensitivity	78.57%	71.43%	78.57%	100.00%	85.19%	100.00%	100.00%	83.15%	100.00%
95% CI	49.20~95.34%	41.90%~91.61%	49.20~95.34%	87.23~100.00%	66.27~100.00%	87.23~100.00%	95.94~100.00%	73.73~90.25%	95.94~100.00%

PSO, post symptom onset.

**Table 6:** Seroconversion pattern based on the detection through GenBody COVID-19 kit.

Sample No.	Symptom Onset/PCR Confirmation Date	Date of Sample Collection for GenBody Kit Analysis	Days PSO	GenBody COVID-19 IgM/IgG		
				IgM	IgG	Remarks
1	2020-03-02	2020-03-08	06	+	+	+
	2020-03-02	2020-03-16	14	+	+	+
	2020-03-02	2020-03-23	21	+	+	+
2	2020-03-09	2020-03-10	01	-	-	-
	2020-03-09	2020-03-17	08	+	+	+
	2020-03-09	2020-03-24	05	+	+	+
	2020-03-09	2020-03-31	22	+	+	+
3	2020-03-10	2020-03-10	00	-	-	-
	2020-03-10	2020-03-25	15	-	+	+
	2020-03-10	2020-03-30	20	-	+	+
4	2020-03-03	2020-03-07	04	+	+	+
	2020-03-03	2020-03-13	10	+	+	+
	2020-03-03	2020-03-19	16	+	+	+
	2020-03-03	2020-03-24	21	+	+	+
5	2020-03-02	2020-03-09	07	+	+	+
	2020-03-02	2020-03-16	14	+	+	+
	2020-03-02	2020-03-25	24	+	+	+

## Data Availability

Data supporting reported results may be provided on reasonable request to the corresponding author.

## Supplementary Materials

The following are available online at https://www.mdpi.com/2075-4418/11/3/537/s1, Table S1: Detection limit of the GenBody COVID-19 kit based on serial dilution of samples.
