# Supplementary material for: Clinical Evaluation of an Immunochromatographic-Based IgM/IgG Antibody Assay (GenBody™ COVI040) for Detection of Antibody Seroconversion in Patients with SARS-CoV-2 Infection
Source: Diagnostics (Basel). 2021 Mar 17;11(3):537. doi: 10.3390/diagnostics11030537 (PMC8002734; doi:10.3390/diagnostics11030537)
Supplement: Supplementary file 1 [file diagnostics-11-00537-s001.pdf]

Supplementary Table S1. Detection limit of the GenBody COVID-19 kit based on serial dilution of samples

Sample	Results	Dilution factor					
		10^{0}	10^{-1}	10^{-2}	10^{-3}	10^{-4}	10^{-5}
1	IgM	+	-	-	-	-	-
	IgG	+	+	+	-	-	-
2	IgM	+	-	-	-	-	-
	IgG	+	+	-	-	-	-
3	IgM	+	-	-	-	-	-
	IgG	+	+	-	-	-	-
4	IgM	+	-	-	-	-	-
	IgG	+	+	-	-	-	-
5	IgM	+	-	-	-	-	-
	IgG	+	+	-	-	-	-
6	IgM	+	+	+	-	-	-
	IgG	+	+	+	-	-	-
7	IgM	+	-	-	-	-	-
	IgG	+	+	+	-	-	-
8	IgM	+	+	-	-	-	-
	IgG	+	+	+	-	-	-
9	IgM	+	+	-	-	-	-
	IgG	+	+	-	-	-	-
10	IgM	+	+	-	-	-	-
	IgG	+	+	+	-	-	-
11	IgM	+	+	-	-	-	-
	IgG	+	+	+	+	+	-
12	IgM	+	+	-	-	-	-
	IgG	+	+	+	+	+	-
13	IgM	+	+	-	-	-	-
	IgG	+	+	-	-	-	-
